# RACK1 promotes tumorigenicity of colon cancer by inducing cell autophagy

**DOI:** 10.1038/s41419-018-1113-9

**Published:** 2018-11-19

**Authors:** Ta Xiao, Wei Zhu, Wei Huang, Shan-Shan Lu, Xin-Hui Li, Zhi-Qiang Xiao, Hong Yi

**Affiliations:** 1 Research Center of Carcinogenesis and Targeted Therapy, Xiangya Hospital, Central South University, Changsha, Hunan 410008 China; 2 Institute of Dermatology, Chinese Academy of Medical Sciences and Peking Union Medical College, Nanjing, Jiangsu 210042 China; 3 Department of Pathology, Xiangya Hospital, Central South University, Changsha, Hunan 410008 China

## Abstract

RACK1 is upregulated in the various types of human cancers, and considered to play a role in the development and progression of human cancer. However, the role and mechanism of RACK in the colon cancer are poorly understood. In this study, we detected RACK1 expression in 63 normal colonic mucosa, 60 colonic inflammatory polyps, 60 colonic adenomas, 180 colon adenocarcinomas, and 40 lymph node metastases by immunohistochemistry, and observed that RACK1 expression was progressively elevated in the carcinogenic process of human colonic epithelium, and RACK1 expressional levels were positively correlated with the malignant degree and lymph node metastasis of colon cancers, and negatively correlated with the patient survival. With a combination of loss-of-function and gain-of-function approaches, we observed that RACK1 promoted colon cancer cell proliferation, inhibited colon cancer cell apoptosis, and enhanced the anchorage-independent and xenograft growth of colon cancer cells. Moreover, we found that RACK1-induced autophagy of colon cancer cells; RACK1-induced autophagy promoted colon cancer cell proliferation and inhibited colon cancer cell apoptosis. Our data suggest that RACK1 acts as an oncogene in colon cancer, and RACK1-induced autophagy promotes proliferation and survival of colon cancer, highlighting the therapeutic potential of autophagy inhibitor in the colon cancer with high RACK1 expression.

## Introduction

The adaptor protein RACK1 (receptor of activated kinase 1) was originally identified as a 36-kDa intracellular receptor for protein kinase C (PKC) isoform βII and is highly conserved among all eukaryotic species^1,2^. As a member of the Trp-Asp (WD) repeat protein family, RACK1 serves as a scaffold protein for many kinases and receptors and plays a pivotal role in a wide range of biological responses, including signal transduction and immune response as well as cell growth, migration, and differentiation^3,4^. RACK1 is ubiquitously expressed in normal tissues, and is found to be upregulated in various kinds of tumors, and considered to play a role in the development and progression of human cancer^5–13^.

In our previous comparative proteomic analysis of normal colonic epithelium between young and old people, we found that RACK1 was downregulated in the aged human colonic epithelium and senescent NIH/3T3 cells, and knockdown of RACK1 by siRNA accelerated the cell senescence^14^. As senescence is characterized by the irreversible loss of proliferation and alongside apoptosis^15–18^, high RACK1 expression may be involved in the pathogenesis of colon cancer. Although other groups have studied the roles of RACK1 in colon cancer, the results are controversial^19–21^. The role and mechanisms of RACK1 in the pathogenesis of colon cancer need to be further elucidated.

Autophagy is a major intracellular degradation system by which cytoplasmic unwanted materials are delivered to and degraded in the lysosome^22^. Autophagic processes can be either constitutive or activated in response to starvation and other stresses. In addition to cellular maintenance, autophagy is involved in many physiological and pathological conditions, such as aging, apoptosis, and cancer^22,23^. The role of autophagy is complex and differs among various types of cancer. Autophagy inhibits tumor initiation and progression in some cancers^24^, and it promotes tumor survival and progression in others^25^, making it as a potential therapeutic target for cancer.

A proteomic study of autophagy-related genes (Atg) complexes found that RACK1 interacts with Atg1, Atg4, Atg14, and Atg18, indicating that RACK1 may act as a scaffold, transiently binding multiple Atg proteins at phagophore assembly sites to promote autophagy^26^. A transcriptomic study of fed and starved control, autophagy-deficient Atg7 and Atg1 null mutant Drosophila also found that RACK1 is an inducer of autophagy and involved in autophagosome formation, and knockdown of RACK1 by siRNA leads to an attenuated autophagic response to starvation^27^. Recent studies indicate that RACK1 participates in the formation of autophagosome biogenesis complex upon its phosphorylation by AMPK at Thr50^28^. Thr50 phosphorylation of RACK1 enhances its direct binding to Vps15, Atg14L, and Beclin1, thereby promoting the assembly of the autophagy-initiation complex and autophagy;^28^ RACK1 also interacts with Atg5 to induce autophagy under the conditions of serum starvation and mTOR inhibition^29^. Although these studies indicate RACK1 as an autophagy inducer in physiology, the role of RACK1 in the regulation of cancer cell autophagy remains unknown.

In the present study, it is of interest to disclose how RACK1 functions in colon cancer. We observed that RACK1 expression was progressively elevated in the carcinogenic process of human colonic epithelium, and was positively correlated with malignant degree and lymph node metastasis of colon cancers, and negatively correlated with patient prognosis; RACK1 enhanced the tumorigenicity of colon cancer cells. Moreover, we found that RACK1-induced colon cancer cell autophagy, and RACK1-induced autophagy promoted colon cancer cell proliferation and inhibited colon cancer cell apoptosis. Our data demonstrate for the first time that RACK1-induced autophagy that might be involved in the pathogenesis of colon cancer.

## Results

### RACK1 expression is progressively increased in the carcinogenic process of human colonic epithelium and negatively correlated with patient prognosis

**Fig. 1:**
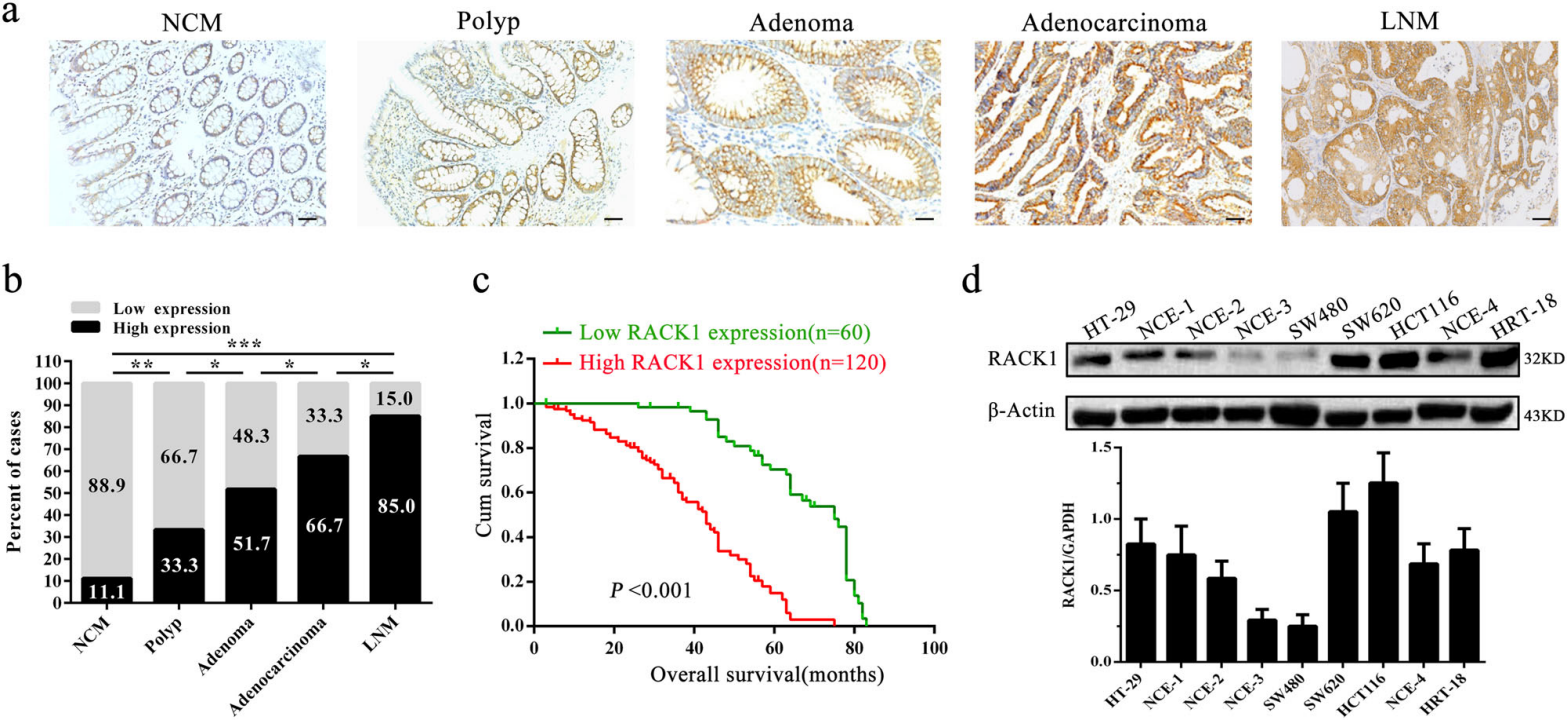
The expression of RACK1 in human colonic epithelial carcinogenic process and colon cancer cell lines and association of RACK1 expression levels with the patient prognosis. **a** A representative result of immunohistochemistry showing the expression of RACK1 in the normal colonic mucosa (NCM), polyp, adenoma, adenocarcinoma, and lymph node metastasis (LNM). Scale bars = 100 μm. **b** Statistical analysis of RACK1 expression in the carcinogenic process of colonic epithelium. *P < 0.01; **P < 0.01; ***P < 0.001. **c** Kaplan–Meier survival analysis for 180 colon cancer patients according to the expression levels of RACK. Patients with high RACK1 expression have a significantly worse overall survival than those with low RACK1 expression. The log-rank test was used to calculate *P*-value. **d** Western blot analysis showing RACK1 levels in the five colon cancer cell lines (HT-29, SW480, SW620, HCT116, and HRT18) and four fresh normal colonic mucosae (NCM)

Till now RACK1 expression in the carcinogenic process of human colonic epithelium has not been investigated, therefore we detected RACK1 expression during the human colon epithelial carcinogenesis including 63 normal colonic mucosa (NCM), 60 colonic inflammatory polyps, 60 colonic adenomas, 180 colon adenocarcinomas, and 40 lymph node metastases (LNM) by immunohistochemical staining. The results showed that RACK1 expression was progressively increased during the colonic epithelial carcinogenesis (Fig. 1a, b; Supplementary Table S1). Survival analysis for colon cancer patients was performed based on the RACK1 levels. The result showed that colon cancer patients with higher RACK1 levels had significantly poorer overall survival (OS) versus patients with lower RACK1 levels (Fig. 1c). A correlation analysis showed significantly positive association of RACK1 expression levels with the malignant degree and lymph node metastasis of colon cancer, but no association of its levels with the patients’ age and gender, and TNM staging (Table 1). These results indicate that high RACK1 expression plays a crucial role in the pathogenesis of colon cancer.

**Table 1:** Correlation between RACK1 expression level and clinicopathological characteristics in colon cancer (n = 180, χ^2^ test)

Variable	*n*	Expression level		*P*
		High (4–6)	Low (0–3)	
*Gender*				
Male	100	64	36	0.3961
Female	80	56	24	
*Age(y)*				
>50	110	72	38	0.6654
≤50	70	48	22	
Differentiation				
High	65	35	30	0.0063
Middle	65	44	21	
Low	50	41	9	
Lymph node metastasis				
N0	103	61	42	0.0167
N1, N2	77	59	18	
Clinical TNM stage				
I, II	108	71	37	0.7469
III, IV	72	49	23	

### RACK1 promotes colon cancer cell proliferation and inhibits colon cancer cell apoptosis

**Fig. 2:**
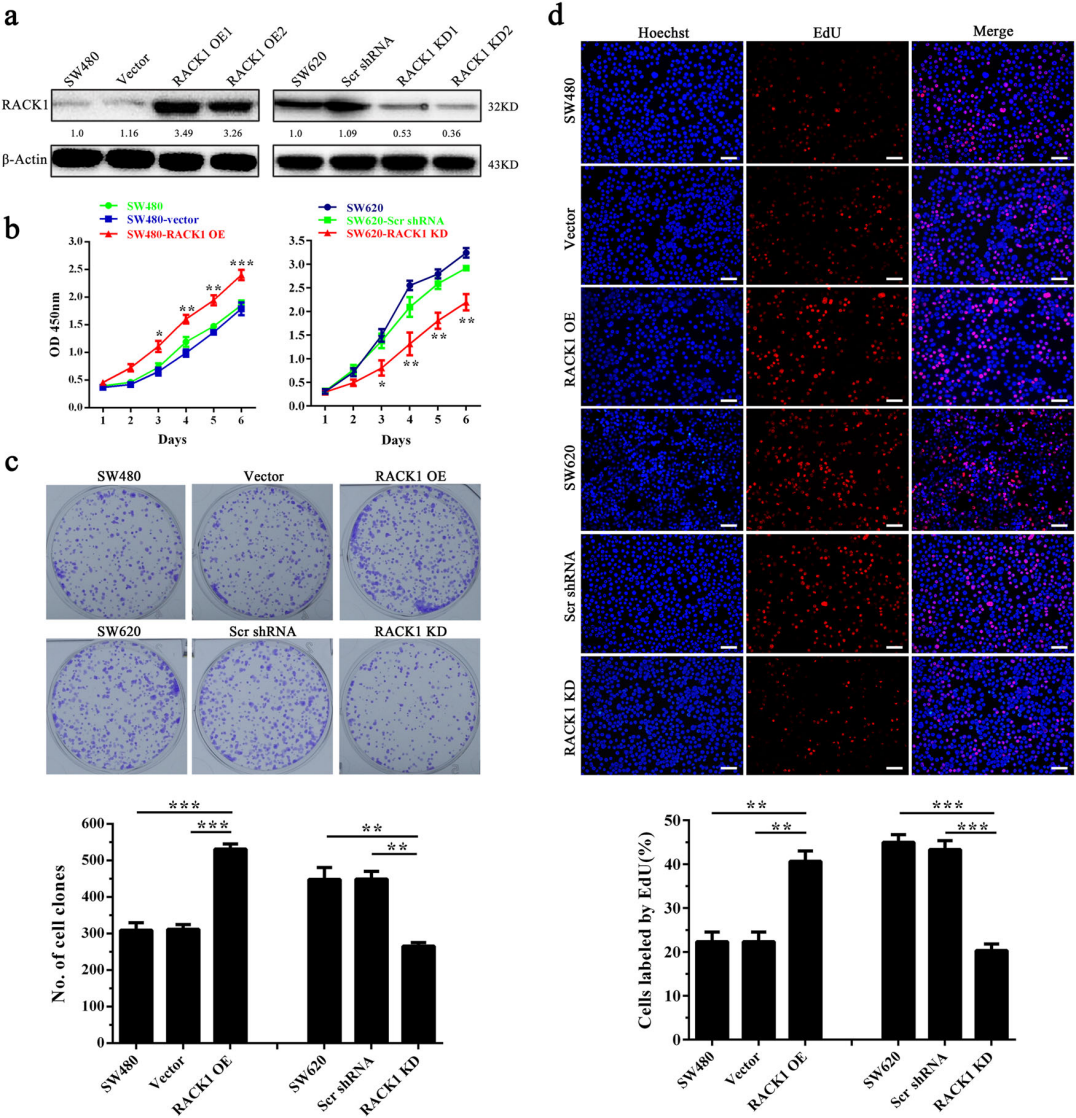
The effects of RACK1 on colon cancer cell proliferation and apoptosis. **a** Western blot analysis showing the expression levels of RACK1 in the SW480 colon cancer cell lines with stable RACK1 overexpression (RACK1 OE), and SW620 colon cancer cell lines with stable RACK1 knockdown (RACK1 KD) and their control cells. Analysis of cell proliferation by CCK-8 (**b**) plate clone formation (**c**) and EdU incorporation (**d**) assay in the SW480-RACK1 OE cells, SW620-RACK1 KD cells and their control cells. Scale bars = 200 μm. Three experiments were done; Means, SDs, and statistical significance are denoted; *P < 0.05; **P < 0.01; ***P < 0.001. Scr, scramble; KD, knockdown; OE, overexpression

**Fig. 3:**
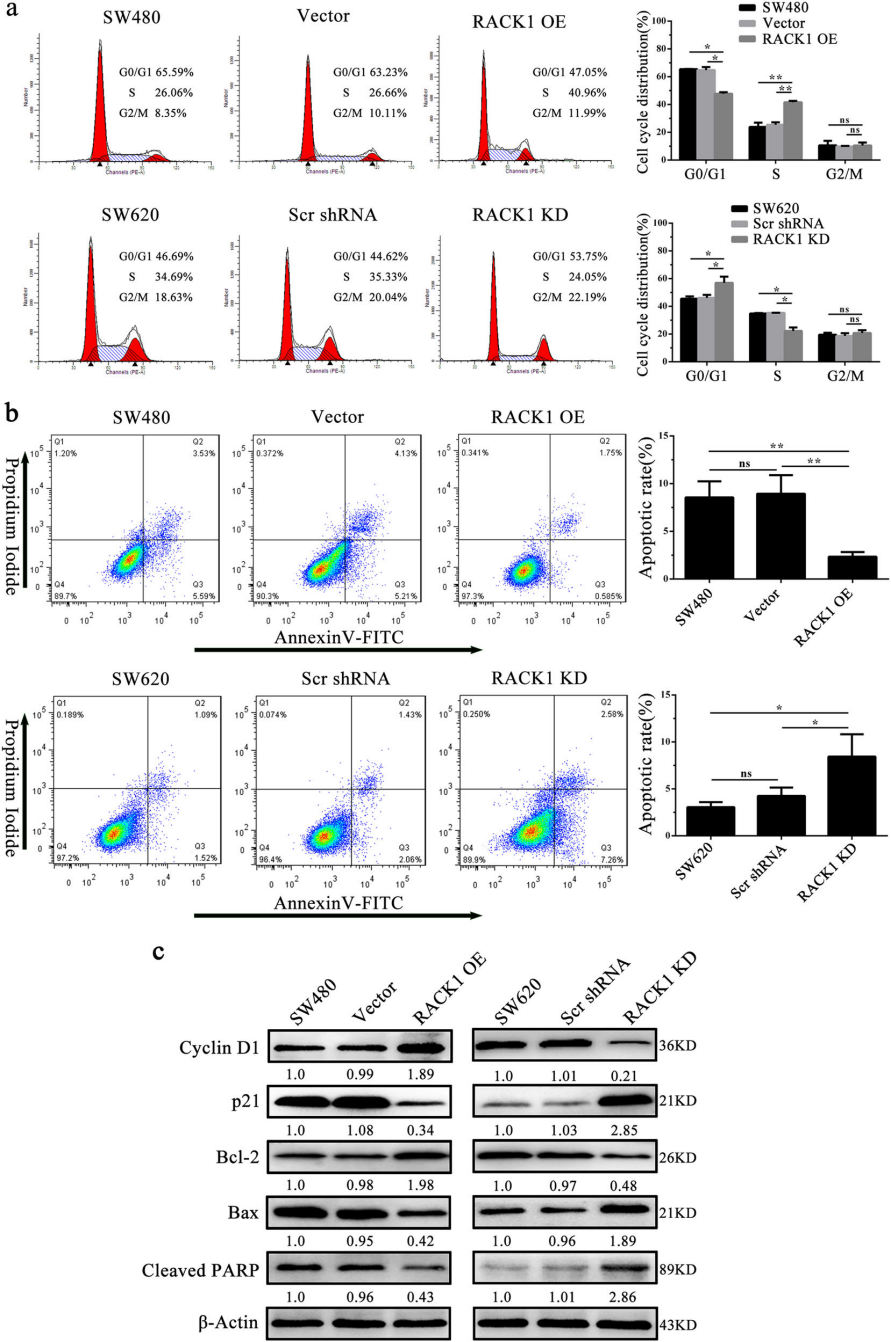
The effects of RACK1 on cell cycle and apoptosis of colon cancer cells. **a** Analysis of cell cycle distribution by flow cytometry in the SW480-RACK1 OE cells, SW620-RACK1 KD cells, and their control cells. **b** Analysis of cell apoptosis by flow cytometry in the SW480-RACK1 OE cells, SW620-RACK1 KD cells and their control cells. **c** Western blot analysis showing the expression levels of cyclin D1, p21, Bcl-2, Bax, and cleaved PARP in the SW480-RACK1 OE cells, SW620-RACK1 KD cells, and their control cells. Three experiments were done; Means, SDs, and statistical significance are denoted; *P < 0.05; **P < 0.01; ns, no significance

We first detected RACK1 expression levels in five colon cancer cell lines (HT-29, SW480, SW620, HCT116, and HRT18) and four fresh NCM by western blot. The results showed that RACK1 expression in the three (SW620, HCT116, and HRT18) of five colon cancer cell lines was obviously higher than that in normal colonic epithelia (Fig. 1d). As SW480 and SW620 cell lines were derived from the primary cancer and lymph node metastasis of the same patient respectively, and RACK1 expression was markedly higher in SW620 cells relative to SW480 cells, we established SW480 colon cancer cell lines with stable RACK1 overexpression (SW480-RACK1 OE), and SW620 colon cancer cell lines with stable RACK1 knockdown (SW620-RACK1 KD) (Fig. 2a). Next, we analyzed the effects of RACK1 levels on clone cancer cell proliferation and apoptosis. CCK-8, plate colony formation, and EdU incorporation labeling assay showed that RACK1 overexpression significantly increased while knockdown significantly decreased colon cancer cell proliferation (Fig. 2b–d). Flow cytometric analysis of cell cycle distribution showed that RACK1 overexpression accelerated G1/S phase progression, whereas RACK1 knockdown blocked cell cycle at G1/S phase (Fig. 3a). Flow cytometric analysis of cell apoptosis showed that RACK1 overexpression significantly decreased while knockdown significantly increased colon cancer cell apoptosis (Fig. 3b). Reexpression of RACK1 in the RACK1 KD colon cancer cells rescued cell proliferation, cell cycle distribution and apoptosis (Supplementary Fig. S1), indicating these phenotypes not due to off-target effects. Moreover, Western blot analysis showed that RACK1 OE upregulated the expression of Bcl-2 and cyclin D, and downregulated the expression of Bax, cleaved PARP and p21, whereas RACK1 KD had the opposite effect on their expression (Fig. 3c), supporting the RACK1-induced-phenotype changes of NPC cell proliferation and apoptosis. Collectively, these results demonstrate that RACK1 promotes colon cancer cell proliferation and inhibits colon cancer cell apoptosis.

### RACK1 increases anchorage-independent growth and in vivo tumorigenicity of colon cancer cells

**Fig. 4:**
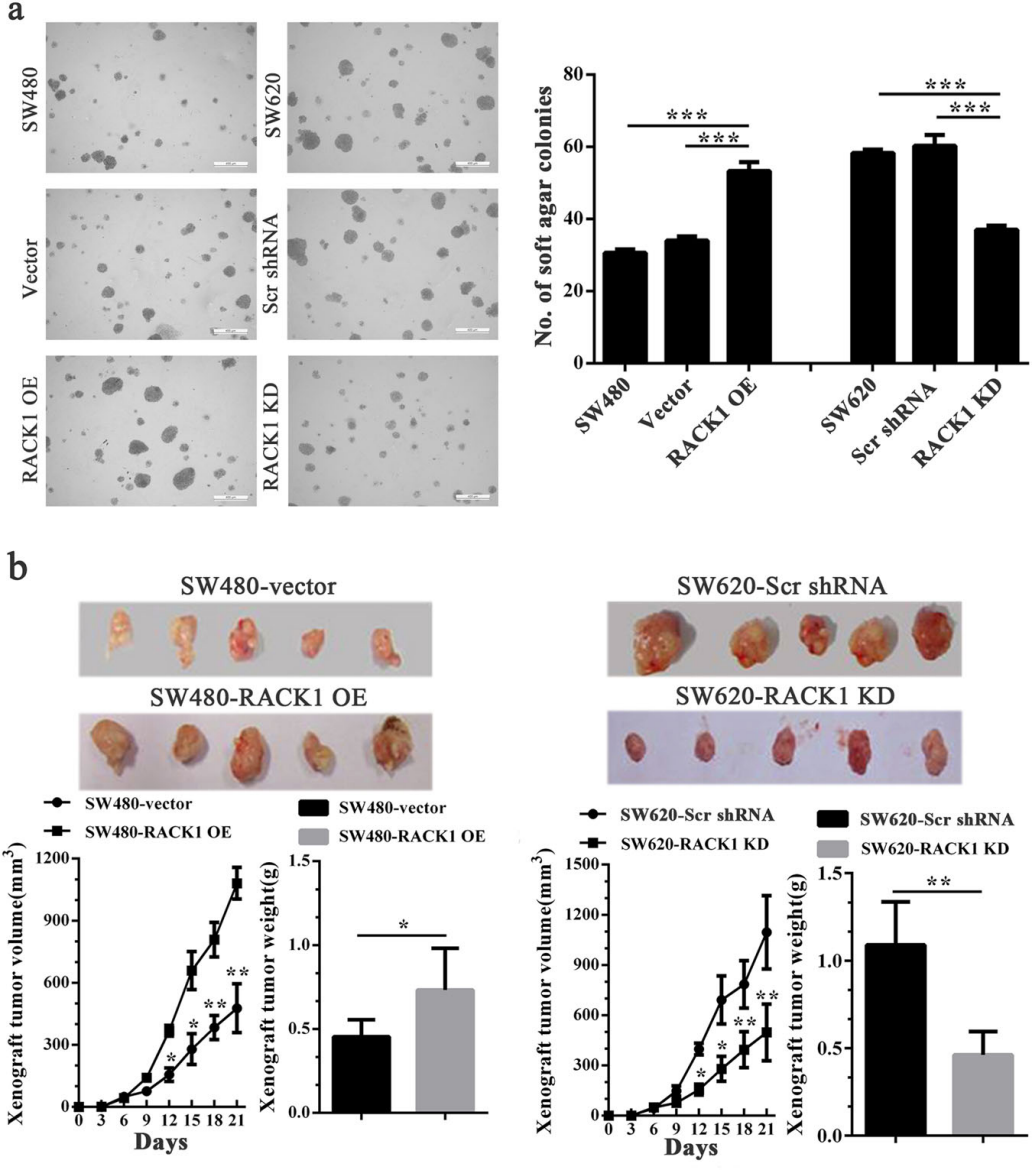
The effects of RACK1 on anchorage-independent and xenograft growth of colon cancer cells. **a** Anchorage-independent colony growth of SW480-RACK1 OE cells, SW620-RACK1 KD cells, and their control cells. (left) Cells were subjected to soft agar colony formation assay, and colonies were photographed under microscope; (right) The histogram showed the average number of soft agar colonies in 10 randomly chosen microscopic fields using a 5 objective. Scale bars = 400 μm. **b** Xenograft growth of SW480-RACK1 OE cells, SW620-RACK1 KD cells, and their control cells. (top) The photography of xenograft tumors after 21 days subcutaneous implantation of cells; (bottom) Growth and weight of the xenograft tumors. *n* = 5 mice per group. Means, SDs, and statistical significance are denoted; *P < 0.05; **P < 0.01; ***P < 0.001

Soft agar colony formation assay and subcutaneous tumor formation experiment in nude mice were performed to determine the effects of RACK1 on the anchorage-independent growth and in vitro tumorigenicity of colon cancer cells respectively. Soft agar colony formation assay showed that RACK1 overexpression significantly increased while knockdown significantly decreased formation ability of soft agar colonies (Fig. 4a), and reexpression of RACK1 in the RACK1 KD colon cancer cells rescued cell anchorage-independent growth (Supplementary Fig. S2), indicating this phenotype not due to off-target effects. Subcutaneous tumor formation experiment showed that RACK1 overexpression significantly increased while knockdown significantly decreased the growth of colon cancer cells in nude mice (Fig. 4b). The results demonstrate that RACK1 increases anchorage-independent growth and in vivo tumorigenicity of colon cancer cells.

### RACK1 induces autophagy in colon cancer cells

**Fig. 5:**
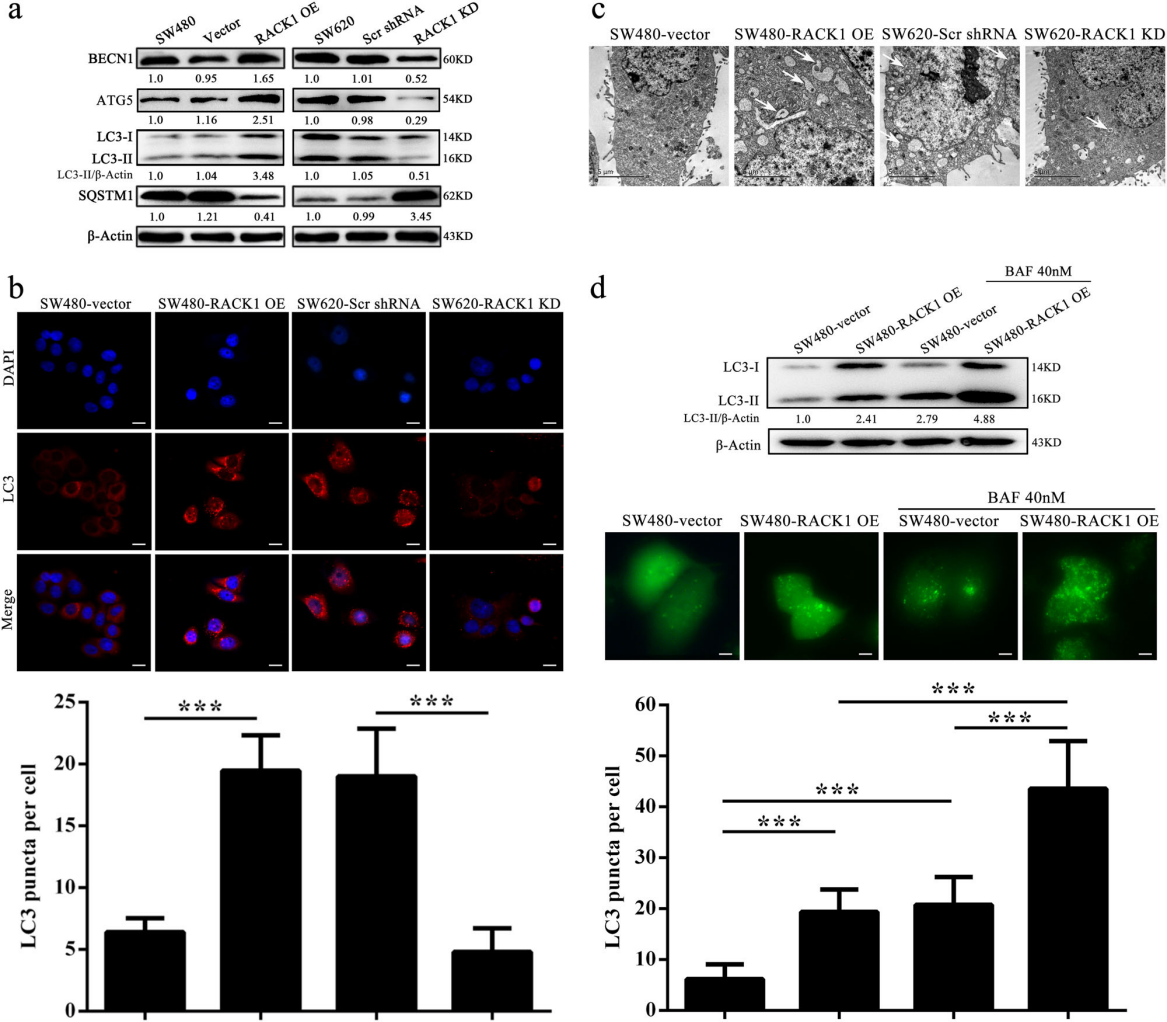
RACK1 induces autophagy and enhances autophagic flux in colon cancer cells. **a** Western blot analysis showing the expression levels of BECN1, ATG5, IC3-II, and SQSTM1 in the SW480-RACK1 OE cells, SW620-RACK1 KD cells, and their control cells. **b** Immunofluorescent staining showing the number of LC3 puncta in the SW480-RACK1 OE cells, SW620-RACK1 KD cells, and their control cells. Cells were stained by indirect immunofluorescence using anti-LC3 antibody, and the number of LC3 puncta per cell was quantified. Scale bars = 100 μm. **c** Electron microscopic examination showing autophagic vacuoles (arrows) in the cytoplasm in the SW480-RACK1 OE cells, SW620-RACK1 KD cells, and their control cells. **d** RACK1 knockdown enhances autophagic flux. (top) Western blot analysis showing the LC3-II levels in the SW480-RACK1 OE cells and control cells treated bafilomycin A1 (BAF). (bottom) The number of EGFP-LC3 puncta in the SW480-RACK1 OE cells and control cells treated BAF. Cells were transfected with 1 μg of EGFP-LC3 plasmid. 24 h after transfection, cells were treated with BAF. 24 h after treatment, EGFP-LC3 puncta was examined by fluorescence microscopy. The number of EGFP-LC3 puncta per cell was quantified. Scale bars = 50 μm. Three experiments were done; Means, SDs, and statistical significance are denoted; ***P < 0.001

Previous studies indicate that RACK1 is an autophagy inducer in physiology^26–29^, but the role of RACK1 in the autophagy of cancer cells is unknown. Therefore we examined the protein levels of Beclin 1 (BECN1, a fundamental gene for autophagy induction)^30^, ATG5, LC3-II (a protein necessary for autophagosome formation) and autophagy-associated protein Sequestosome-1 (SQSTM1) in the colon cancer cell lines with RACK1 expression changes to determine whether RACK1 regulates autophagy of colon cancer cells. As shown in Fig. 5a, a significant increase in BECN1, ATG5, and LC3-II along with reduction of SQSTM1 was found in the RACK1 OE SW480 cells, whereas a significant decrease in BECN1, ATG5, and LC3-II along with elevation of SQSTM1 was observed in the RACK1 KD SW620 cells, as compared to their control cells. Immunofluorescent staining showed that the puncta-staining pattern of LC3 was significantly increased in the RACK1 OE SW480 cells, whereas was significantly decreased in the RACK1 KD SW620 cells, as compared to their control cells (Fig. 5b). Moreover, using an electron microscopy technique, we observed that the numbers of autophagic vacuoles (AVs) were markedly increased in the RACK1 OE SW480 cells, whereas were markedly decreased in the RACK1 KD SW620 cells, as compared to their control cells(Fig. 5c). These results demonstrate that RACK1 induces autophagy in the colon cancer cells.

LC3-I is in turn lapidated to LC3-II, which then associates with autophagosome membranes^31^. LC3-II can accumulate due to increased upstream autophagosome formation or impaired downstream autophagosome–lysosome fusion. To distinguish between these two possibilities, we assayed LC3-II in the presence of bafilomycin A1 (BAF) by western blot, which blocks downstream autophagosome–lysosome fusion^32^. As shown in Fig. 5d, BAF further increased the levels of LC3-II induced by RACK1 overexpression in the colon cancer cells. Besides, RACK1 OE colon cancer cells were transfected with a plasmid-expressing EGFP fused with LC3 (EGFP-LC3) for 24 h and BAF treatment for an additional 24 h, thereafter the number of EGFP-LC3 puncta was examined. We observed that BAF further increased the number of EGFP-LC3 puncta induced by RACK1 OE in the colon cancer cells (Fig. 5d). The results strongly indicate that RACK1 overexpression enhances the autophagic flux of colon cancer cells.

### RACK1-induced autophagy enhances colon cancer cell proliferation and inhibits colon cancer cell apoptosis

**Fig. 6:**
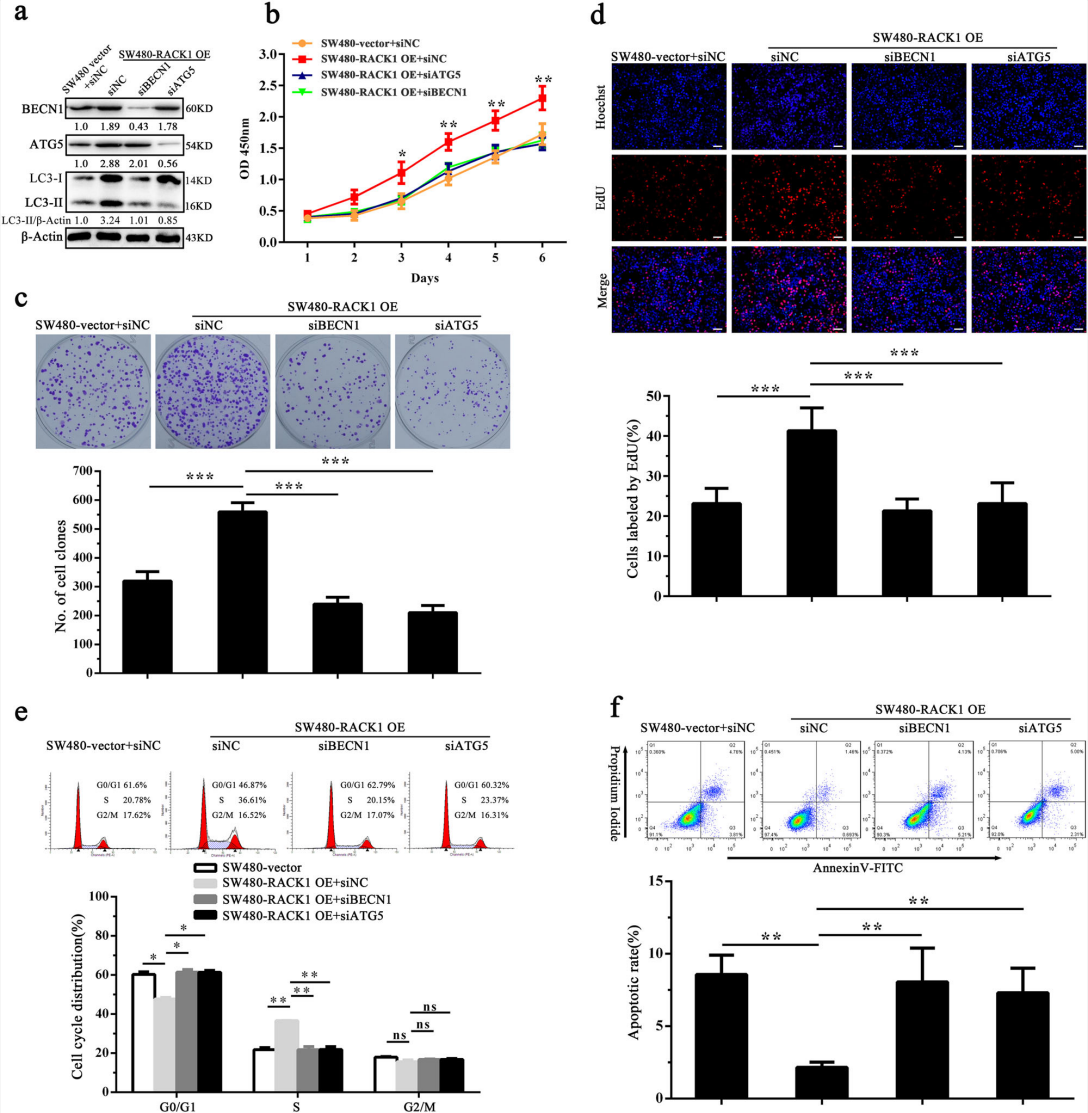
The effect of BECN1 and ATG5 siRNAs on proliferation and apoptosis of RACK1 OE colon cancer cells. **a** Western blot analysis showing the expression levels of BECN1, ATG5, and LC3-II in the SW480-RACK1 OE cells transfected with BECN1 or ATG5 siRNA and control cells. Analysis of cell proliferation by CCK-8 (**b**), plate clone formation(**c**) and EdU incorporation (**d**) assay in the SW480-RACK1 OE cells transfected with BECN1 or ATG5 siRNA and control cells. Analysis of cell cycle distribution (**e**) and cell apoptosis (**f**) by flow cytometry in the SW480-RACK1 OE cells transfected with BECN1 or ATG5 siRNA and control cells. Three experiments were done; Means, SDs, and statistical significance are denoted; *P < 0.05; **P < 0.01; ***P < 0.001; ns, no significance. Scale bars = 200 μm. siBECN1, BENC1 siRNA; siATG5, ATG5 siRNA; siNC, negative control siRNA

**Fig. 7:**
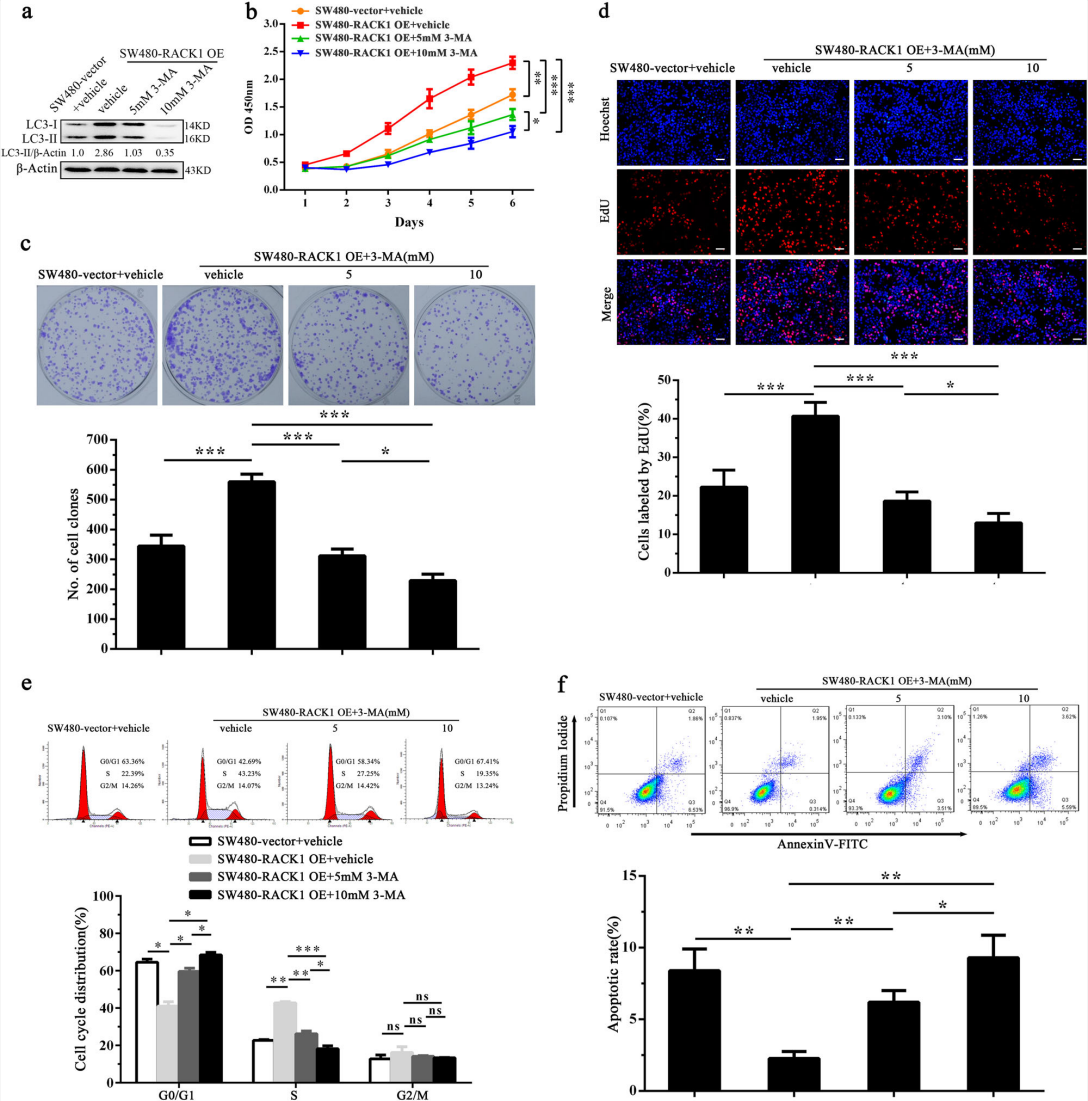
The effect of 3-MA on proliferation and apoptosis of RACK1 OE colon cancer cells. **a** Western blot analysis showing the expression levels of LC3-II in the SW480-RACK1 OE cells treated with 3-methyladenine (3-MA). Analysis of cell proliferation by CCK-8 (**b**), plate clone formation (**c**) and EdU incorporation (**d**) assay in the SW480-RACK1 OE cells treated with 3-MA and control cells. Analysis of cell cycle distribution (**e**) and cell apoptosis (**f**) by flow cytometry in the SW480-RACK1 OE cells treated with 3-MA and control cells. Three experiments were done; Means, SDs, and statistical significance are denoted; *P < 0.05; **P < 0.01; ***P < 0.001; ns, no significance. Scale bars = 200 μm

Previous studies have indicated that autophagy modulates the proliferation and apoptosis of cancer cells^33–37^. To determine whether RACK1-induced autophagy promotes colon cancer cell proliferation and inhibits colon cancer cell apoptosis, we used siRNA against ATG5 or BECN1 to inhibit autophagy in the RACK1 OE colon cancer cells (Fig. 6a), and analyzed the changes of cells proliferation and apoptosis. CCK-8, plate colony formation, and EdU incorporation labeling assay showed that both siRNAs significantly decreased RACK1 OE colon cancer cells proliferation as compared with control siRNA (Fig. 6b–d). Flow cytometric analysis showed that both siRNAs blocked RACK1 OE colon cancer cell cycle at G1/S phase (Fig. 6e), and significantly increased RACK1 OE colon cancer cell apoptosis as compared to control siRNA (Fig. 6f). Moreover, we used 3-methyladenine (3-MA), a class III phosphatidylinositol 3-kinase (PtdIns3K) inhibitor, to inhibit autophagy in the RACK1 OE colon cancer cells (Fig.7a), and analyzed the changes of cells proliferation and apoptosis. The results showed that 3-MA also decreased cells proliferation (Fig. 7b–d), blocked cell cycle at G1/S phase (Fig. 7e) and increased cells apoptosis (Fig. 7f) in the RACK1 OE colon cancer cells. The results suggest that RACK1-induced-autophagy promotes colon cancer cell proliferation, inhibits colon cancer cell apoptosis.

## Discussion

Although RACK1 is considered to play a role in the development and progression of human cancer^5–13^, the role and mechanisms of RACK1 in the pathogenesis of human colon cancer are poorly understood^19–21^. In the present study, we found that RACK1 expression was progressively increased during the colonic epithelial carcinogenesis; RACK1 expressional levels were positively correlated with differentiation degree and lymph node metastasis of colon cancer, and negatively correlated with the patient survival. These results indicate that RACK1 plays a role in colon cancer.

Our previous study showed that RACK1 expression was downregulated in the aged human colonic epithelium and senescent NIH/3T3 cells, and knockdown of RACK1 accelerated NIH/3T3 cell senescence^14^. Senescence acts as a powerful tumor suppressor to prevent the proliferation of damaged cells, and protects cells expressing activated oncogenes in vivo from becoming neoplastic and malignant^15,38,39^. Therefore high RACK1 expression may be involved in the pathogenesis of colon cancer by inhibiting cells senescence. As senescence is characterized by the irreversible loss of proliferation and alongside apoptosis^15–18^, we investigated the effects of RACK1 on the proliferation and apoptosis of colon cancer cells, and observed that RACK1 overexpression significantly increased while knockdown significantly decreased colon cancer cell proliferation; RACK1 overexpression significantly decreased while knockdown significantly increased colon cancer cell apoptosis; RACK1 overexpression accelerated G1/S phase progression, whereas RACK1 knockdown blocked cell cycle at G1/S phase in the colon cancer cells. Moreover, we observed that RACK1 overexpression significantly increased while knockdown significantly decreased the anchorage-independent and xenograft growth of colon cancer cells. Together, our results indicate that high RACK1 expression promotes tumorigenicity of colon cancer cells possibly through increasing colon cancer cell proliferation and inhibiting colon cancer cell apoptosis.

Previous studies suggest that RACK1 is an autophagy inducer in physiology^26–29^, but the role of RACK1 in the autophagy of cancer cells is unknown. Therefore, we investigated the regulation of RACK1 on autophagy of colon cancer cells. We found that high RACK1 expression promoted colon cancer cell autophagy. Moreover, we observed that RACK1 overexpression enhanced the autophagic flux of colon cancer cells. To our knowledge, it is first time reported that RACK1 regulates cancer cell autophagy. Previous studies have indicated that autophagy modulates cancer cell proliferation and apoptosis^33–37^. Therefore, we investigated the association of RACK1-induced autophagy with the proliferation and apoptosis of colon cancer cells. We observed that both chemical and genetic inhibition of autophagy decreased cell proliferation and increased cell apoptosis in the colon cancer cells with RACK1 overexpression, indicating that RACK1-induced autophagy promotes colon cancer cell proliferation and inhibits colon cancer cell apoptosis, which might be related to tumorigenicity of RACK1.

How does RACK1 regulate colon cancer cell autophagy? We observed that RACK1 overexpression upregulated while RACK1 knockdown downregulated phospho-JNK level in the colon cancer cells (Supplementary Fig. S3). It has been reported that RACK1 activates JNK-signaling pathway in the cancer cells^40–42^. There have been numerous reporters on JNK-regulating autophagy in cancer cells^43–45^. Therefore, we presume that RACK1 induces colon cancer cell autophagy by activating JNK signaling pathway.

In summary, our data demonstrate that: (a) RACK1 expression is progressively increased during the colonic epithelial carcinogenesis, and is positively correlated with malignant degree and lymph node metastasis of colon cancer, and negatively correlated with the patient survival; (b) RACK1 enhances tumorigenicity of colon cancer cells; (c) RACK1 promotes autophagy of colon cancer cells; (d) RACK1-induced autophagy increases colon cancer cell proliferation and inhibits colon cancer cell apoptosis. Our data demonstrate that RACK1 acts as an oncogene in colon cancer, and suggest that RACK1-induced autophagy might be involved in the pathogenesis of colon cancer.

## Materials and methods

### Patients and tissue samples

The formalin-fixed and paraffin-embedded archival tissue specimens of 180 colon cancers, and 40 LNMs paired with primary cancers between Jan 2009 and Dec 2012 were obtained from the Xiangya Hospital of Central South University, China. The patients underwent radical surgery, and no cases received preoperative chemotherapy and radiotherapy. The clinicopathological data of patients were reviewed, including gender, age, differentiation degree, lymph node metastasis, and TNM stage (Table 1). We also acquired 63 normal colonic mucosa (NCM), 60 colon inflammatory polyps and 60 colon adenomas in the same period. All specimens were subjected to HE staining, and the diagnosis was confirmed by two pathologists. Staging of colon cancer was based on pathological findings, according to the American Joint Committee on Cancer (AJCC). The patients were followed up, and the follow-up period at the time of analysis was more than 70 months. OS was defined as the time from the initiation of operation to the date of cancer-related death or when censured at the latest date if patients were still alive. OS was calculated using the log-rank test with a Kaplan–Meier curve.

In addition, four cases of fresh NCM were obtained from healthy male volunteers at Xiangya Hospital of Central South University, China, with an informed consent. Only the superficial layer of the colonic mucosa was collected under endoscope to ensure that the tissue samples consisted mostly of epithelium. The tissues were immediately frozen in liquid nitrogen until Western blot analysis.

### Cell lines

Human colon cell lines (HT-29, SW480, SW620, HCT116, and HRT18) were purchased from the American Type Culture Collection (ATCC) in 2010, maintained in our laboratory, and cultured with RPMI-1640 medium supplemented with 10% fetal bovine serum (Life Technologies) at 37 °C in 5% CO_2_. The cell lines were routinely tested for presence of mycoplasma with 4,6-diamidino-2-phenylindole staining, and were mycoplasma free.

Antibodies and reagents. Antibodies against RACK1 (sc-17754), Cyclin D1 (sc-450), Bax (sc-20067), cleaved PARP (sc-56196), Bcl-2 (sc-509) and JNK-2 (sc-7345) were purchased from Santa Cruz. Antibodies against SQSTM1 (ab207305), BECN1 (ab62557) and LC3-II (ab192890) were purchased from Abcam. Antibodies against LC3-I/II antibody (#4108) and phospho-JNK-1/2(#9255) were purchased from CST. Horseradish peroxidase-conjugated goat anti-rabbit (#A24531) and anti-mouse IgG antibodies (#A24512) were purchased from Life Technologies. Bafilomycin A1 (B1793) and 3-methyladenine (M9281) were purchased from Sigma-Aldrich.

### Establishment of colon cancer cell lines with stable RACK1 knockdown and overexpression

Lentiviral GV248 vector expressing RACK1 shRNA or scramble non-target shRNA, and Lentiviral GV358 vector expressing RACK1 and control vector GV358 were established by Genechem Co. (Shanghai, China), and confirmed by sequencing. The target for human lentiviral shRNA was 5′-CAGGGATGAGACCAACTATGG-3′, the knockdown efficiency of which has been validated^46^. SW620 and SW480 cells were infected with the lentiviral particles according to the manufacturer’s instructions, respectively, and then selected using puromycin for 2 weeks. Colon cancer cell lines with stable knockdown or overexpression of RACK1 and control cell lines were obtained.

### Gene silencing by siRNAs

siRNAs against BECN1 (sc-29797) and ATG5 (sc-41445), purchased from Santa Cruz, were used to knockdown the BECN1 and ATG5 expression in the indicated cells in according to the manufacturer’s protocol. The control siRNA (sc-37007) was used as a control.

### Immunofluorescent staining

immunofluorescent staining was performed as described previously by us^47,48^. Briefly, 2 × 10^3^ cells were plated into chamber slides (Millipore) and cultured with RPMI-1640 medium containing 10% FBS for 12 h. Cells were fixed with 4% paraformaldehyde at RT for 15 min, and then cell membranes were permeabilized with 0.1% Triton 100 at RT for 20 min. Cells were washed with 1 × PBS and blocked with 5% bull serum albumin(BSA) in PBS for 1 h. Then cells were incubated with primary antibody against LC3 overnight at 4 °C. After washing with 1 × PBS for three times, cells were incubated with secondary antibodies conjugated with Alexa Fluor 594 for 1 h. The slides were washed three times with 1 × PBS, counterstained with DAPI, mounted and stored at 4 °C under dark conditions. Autophagy was quantified by quantification of LC3 dots per cell using Leica DMI4000 fluorescence microscope. The average number of LC3 dot per cell was counted with at least 100 cells for each cell line.

### Quantification of EGFP-LC3 puncta

The cells were transfected with EGFP-LC3 expression plasmid using Lipofectamine 2000 (Invitrogen). Afterward, cells were fixed in 4% paraformaldehyde at RT for 20 min, and washed twice with PBS. Autophagy was quantified by quantification of EGFP-LC3 puncta per cell using Leica DMI4000 fluorescence microscope. The average number of EGFP-LC3 puncta per cell was counted with at least 100 cells for each cell line.

### Transmission electron microscopy

The cells were fixed in the fixative buffer (2.5% glutaraldehyde, 4% paraformaldehyde, 8 µM calcium chloride, 0.1 M sodium cacodylate, pH 7.4) overnight at 4 °C. After a buffer wash, the cells was post-fixed in 1% OsO_4_ with 1.5% potassium ferricyanide in 0.1 M cacodylate for 1 h at 4 °C, dehydrated in graded ethanols, and then embedded in epoxy resin. The cells were ultra-thin sectioned 70 nm in thickness, mounted on copper slot grids coated with parlodion and stained again with uranyl acetate and lead citrate. The sections were examined using a Philips CM100 electron microscope at 60 kV. Images were recorded digitally using a Hamamatsu ORCA-HR digital camera system.

### Cell counting Kit-8 (CCK-8) assay

Cell proliferation was measured using a CCK-8 kit. Briefly, the cells were plated at 1 ×1 0^4^ cells per well in 96-well tissue culture plates, and grew for 7 d. Every 24 h, 10 µl CCK-8 reagent (Beyotime, Nanjing, China) was added to every well, and incubated for 4 h. The absorbance of each well was read with a Bio-Tek Instruments EL310 Microplate Autoreader at 450 nm. CCK-8 assay was performed three times in triplicate.

### 5-ethynyl-2′-deoxyuridine (EdU) incorporation assay

Cell proliferation was measured using EdU assay. Briefly, the cells were cultured in chamber slides (Millipore; 2 × 10^4^ cells/well). 48 h after culture, the cells were treated with 50 µM EdU (RiboBio, Guanzhou, China) for an additional 2 h at 37 °C, and then were fixed with 4% formaldehyde for 30 min, followed by addition of 200 µl glycine (2 mg/ml; Amresco). After 5 min, the cells were incubated with 0.5% Triton X-100 (Sigma-Aldrich) for 10 min at room temperature. Following washing with PBS for 5 min, 1 × Apollo reaction reagent (RiboBio) was added and incubated at RT in the dark for 30 min, and then the cells were stained with 200 µl Hoechst 33342 (5 µg/ml; Sigma-Aldrich) for an additional 30 min in the dark. Cells labeled and unlabeled by EdU were counted under a Leica DMI4000 microscope, and pictures were taken.

### Anchorage-dependent and independent colony formation assay

Plate colony formation and soft agar colony formation assay were done as previously described by us^47,49^.

### Flow cytometry analysis

Analyses of cell cycle and apoptosis by flow cytometry were done as previously described by us^47,49^.

### Tumor formation assay in nude mice

Nude male mice that were 4-weeks old were obtained from the Laboratory Animal Center of Central South University (Changsha, China) and were maintained under specific pathogen-free conditions. For tumor formation experiment, 5 × 10^6^ cells resuspended in 200 μl of medium without serum were subcutaneously injected into the flanks of mice (*n* = 5 mice each). The mice were monitored daily for palpable tumor formation, and tumor volume (in mm^3^) was measured by a Vernier caliper every 3 days and calculated by using the modified ellipse formula (volume = length × width^2^/2). After 3 weeks, the mice were killed by cervical dislocation, and their tumors were excised, and weighted.

### Western blot

Proteins were extracted from cells and tissues. An equal amount of protein in each sample was subjected to SDS-PAGE separation, followed by blotting onto a PVDF membrane. After blocking, blots were incubated with primary antibody overnight at 4 °C, followed by incubation with HRP-conjugated secondary antibody for 1 h at room temperature. The signal was visualized with an enhanced chemiluminescence detection reagent (Millipore). β-actin was detected as a loading control. The blot bands were quantified using Gel-Pro analyzer version 6.0.

### Immunohistochemistry

Immunohistochemistry and staining evaluation of RACK1 were performed on the formalin-fixed and paraffin-embedded tissue sections as described previously by us^47,49^.

### Statistical analyses

All experiments were carried out at least three times. Data were presented as the mean ± standard deviation. Statistical analysis was conducted using SPSS 22.0 statistical software package. For comparisons between two groups, a Student *t*-test, chi-square test or Wilcoxon and Mann Whitney test were used, and for analysis with multiple comparisons, Oneway ANOVA test was used. Survival curves were obtained by using Kaplan–Meier method, and comparisons were made by using log-rank test. All statistical tests were 2-sided. *P*-values less than 0.05 were considered to be statistically significant.

### Ethics statement

This study was approved by the Institute Research Ethics Committee of Xiangya Hospital, Central South University, China. All animal experiments were undertaken in accordance with the Guide for the Care and Use of Laboratory Animals of Central South University, with the approval of the Scientific Investigation Board of Central South University. As only archived tumor specimens were included in this study, the ethics committee waived the need for consent and patient records/information were analyzed anonymously.

## Electronic supplementary material

Supplemental Table S1 and Supplemental Figure S1, 2 and 3
