# Supplementary material for: RACK1 promotes tumorigenicity of colon cancer by inducing cell autophagy
Source: Cell Death Dis. 2018 Nov 19;9(12):1148. doi: 10.1038/s41419-018-1113-9 (PMC6242835; doi:10.1038/s41419-018-1113-9)
Supplement: Supplementary file 1 — Supplemental Table S1 and Supplemental Figure S1, 2 and 3 [file 41419_2018_1113_MOESM1_ESM.pdf]

Supplementary Table S1. The expression level of RACK1 in the colon epithelial carcinogenic process

Expression level	NCM (n, %)	Polyp (n, %)	Adenoma (n, %)	Adenocarcinoma (n, %)	LNM (n, %)
Low (0-3)	56(88.9)	40(66.7)	29(48.3)	60(33.3)	6(15.0)
High (4-6)	79(11.1)	20(33.3)	31(51.7)	120(66.7)	34(85.0)
Sum	63	60	60	180	40

NCM, normal colon mucosa; LNM, lymph node metastasis

χ^2 test. The overall difference: P <0.001; NCM vs. Polyp: P <0.01; Polyp vs. Adenoma: P <0.05; Adenoma vs. Adenocarcioma: P <0.05; Adenocarcioma vs. LNM: P <0.05.

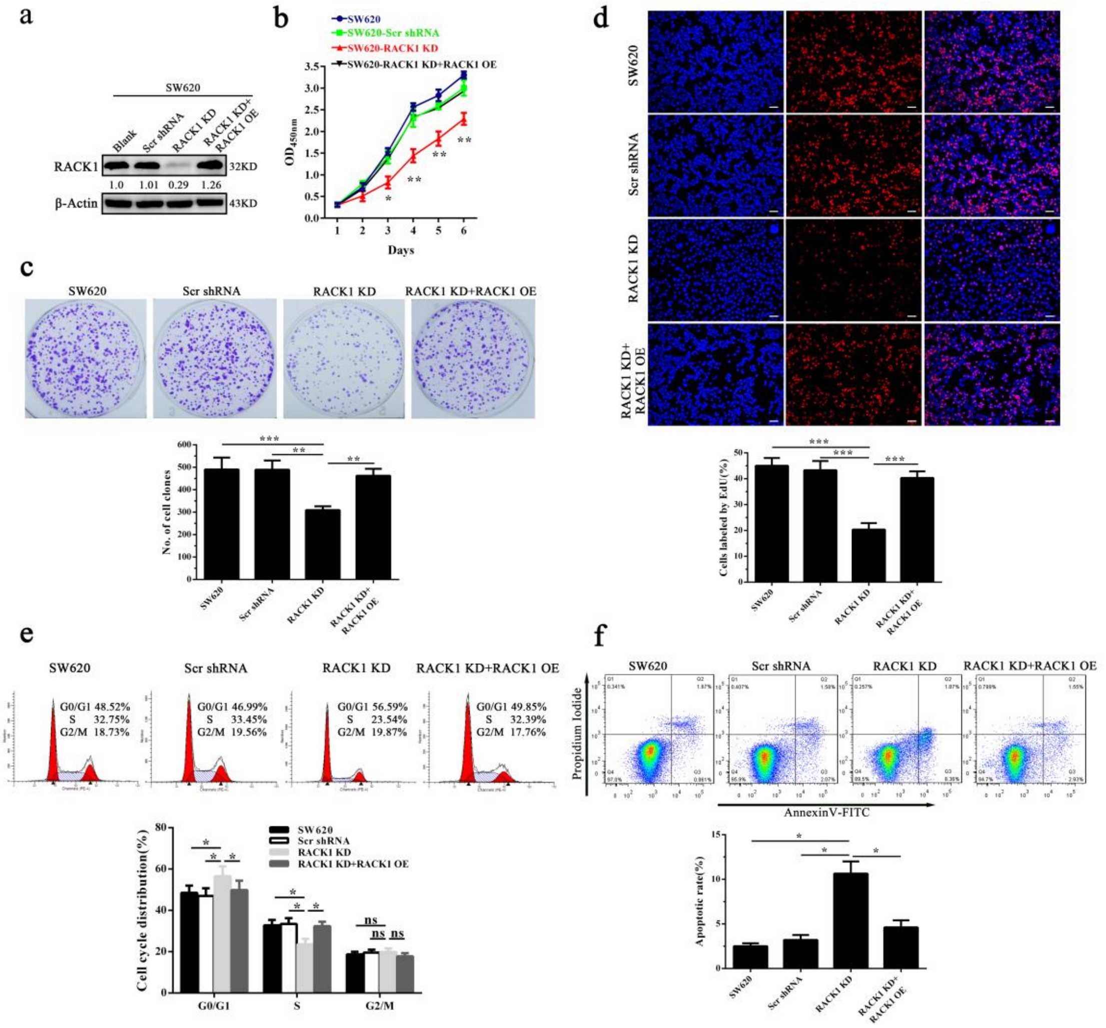

Supplementary Figure S1. Reexpression of RACK1 rescues cell proliferation and apoptosis in the RACK1 KD SW620 cells. (**a**) Western blot analysis showing the expression levels of RACK1 in the SW620-RACK1 KD cells [shRNA targeting the 3'UTR (5'-GTAAAGCTCTGCCATAAACTT-3') of human RACK1 transcript] transfected with lentiviral vector expressing RACK1 ORF and its control cells. Analysis of cell proliferation by CCK-8 (**b**), plate clone formation (**c**) and EdU incorporation (**d**) assay in the SW620-RACK1 KD cells transfected with lentiviral vector expressing RACK1 ORF and its control cells. Analysis of cell cycle distribution (**e**) and cell apoptosis (**f**) by flow cytometry in the SW620-RACK1 KD cells transfected with lentiviral vector expressing RACK1 ORF and its control cells. Three experiments were done; Means, SDs, and statistical significance are denoted; *, *P*<0.05; **, *P*<0.01; ***, *P*<0.001; ns, no significance. Scale bars = 200μm.

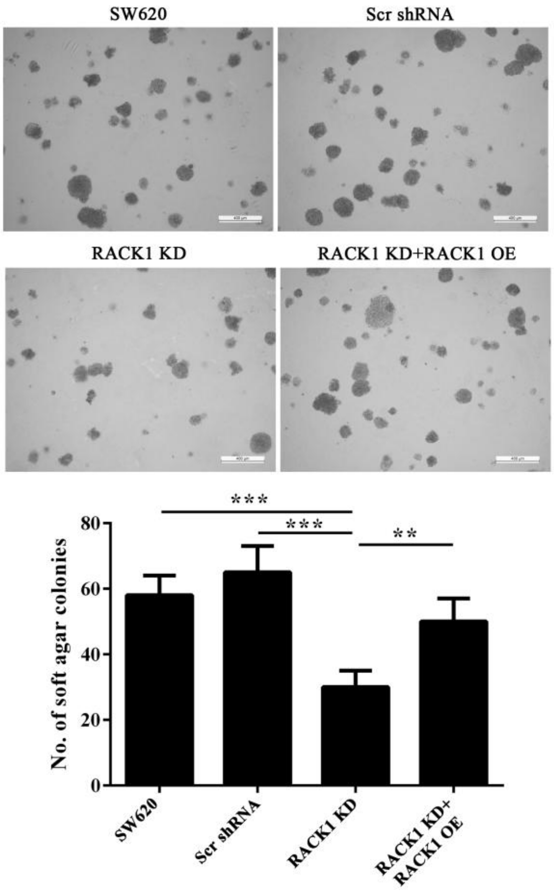

Supplementary Figure S2. Reexpression of RACK1 rescues cell anchorage independent growth in the RACK1 KD SW620 cells. Analysis of cell anchorage independent growth by soft agar colony formation assay in the SW620-RACK1 KD cells transfected with lentiviral vector expressing RACK1 ORF and its control cells. Three experiments were done; Means, SDs, and statistical significance are denoted; **, $P<0.01$; ***, $P<0.001$. Scale bars = 400μm.

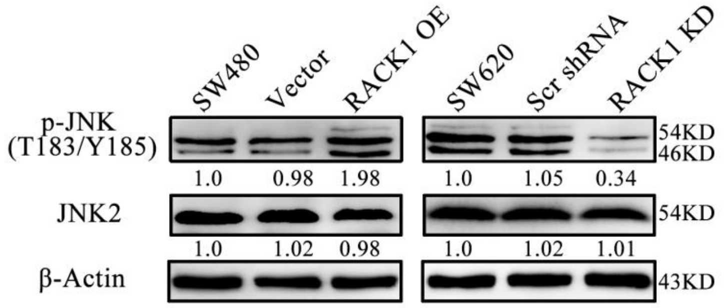

Supplementary Figure S3. RACK1 activates JNK signaling in the colon cancer cells. Western blot analysis showing the expression levels of phospho-JNK-1/2 and JNK-2 in the SW480-RACK1 OE cells, SW620-RACK1 KD cells and their control cells.
